# Self-association of the glycopeptide antibiotic teicoplanin A2 in aqueous solution studied by molecular hydrodynamics

**DOI:** 10.1038/s41598-023-28740-8

**Published:** 2023-02-03

**Authors:** Taewoo Chun, Jacob Pattem, Richard B. Gillis, Vlad T. Dinu, Gleb E. Yakubov, Anthony P. Corfield, Stephen E. Harding

**Affiliations:** 1 National Centre for Macromolecular Hydrodynamics, University of Nottingham, Sutton Bonington, LE12 5RD UK; 2 Biomaterials Group, School of Biosciences, University of Nottingham, Sutton Bonington, LE12 5RD UK; 3 ARC Centre of Excellence in Plant Cell Walls, School of Chemical Engineering, The University of Queensland, St. Lucia, Brisbane, QLD 4072 Australia

**Keywords:** Biochemistry, Biotechnology

## Abstract

The natural glycopeptide antibiotic teicoplanin is used for the treatment of serious Gram-positive related bacterial infections and can be administered intravenously, intramuscularly, topically (ocular infections), or orally. It has also been considered for targeting viral infection by SARS-CoV-2. The hydrodynamic properties of teicoplanin A2 (*M*_1_ = 1880 g/mol) were examined in phosphate chloride buffer (pH 6.8, *I* = 0.10 M) using sedimentation velocity and sedimentation equilibrium in the analytical ultracentrifuge together with capillary (rolling ball) viscometry. In the concentration range, 0–10 mg/mL teicoplanin A2 was found to self-associate plateauing > 1 mg/mL to give a molar mass of (35,400 ± 1000) g/mol corresponding to ~ (19 ± 1) mers, with a sedimentation coefficient *s*_20, w_ =  ~ 4.65 S. The intrinsic viscosity [$\eta$] was found to be (3.2 ± 0.1) mL/g: both this, the value for *s*_20,w_ and the hydrodynamic radius from dynamic light scattering are consistent with a globular macromolecular assembly, with a swelling ratio through dynamic hydration processes of ~ 2.

## Introduction

**Figure 1:**
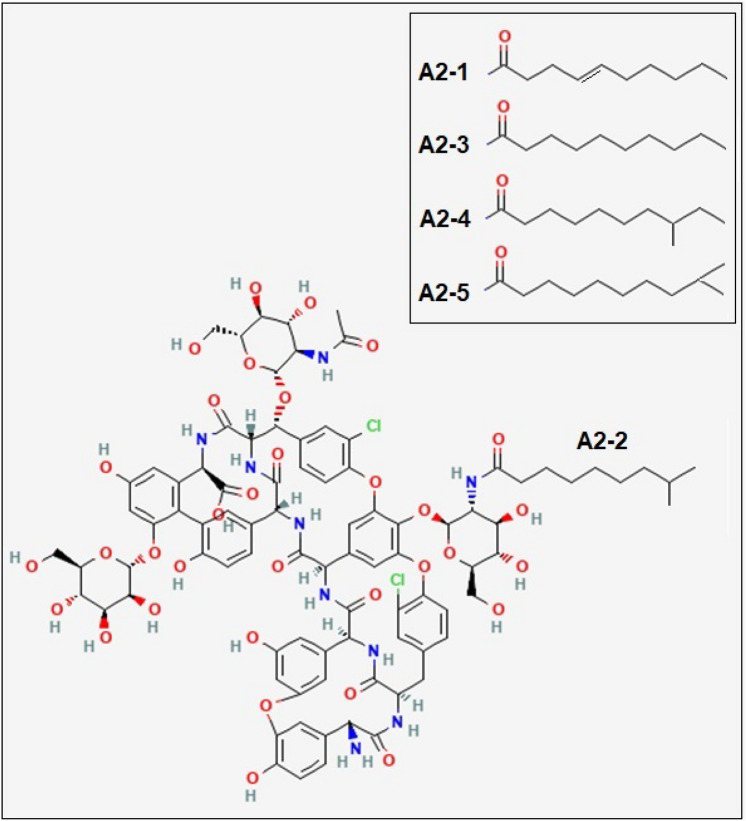
Structure of teicoplanin (adapted from the National Institute for Health/ National Center of Biotechnology Information^36^ based on an original structure given by F. Parenti^37^).

Teicoplanin is a member of the glycopeptide antibiotic family, such as vancomycin, to treat severe bacterial infections. This glycopeptide antibiotic was first extracted from *Actinoplanes teichomyceticus*, which was discovered in 1978 from an Indian soil sample^1^. Its main chemical structure (Fig. 1) is a heptapeptide with three monosaccharide residues: α-D-mannose, *N*-acetyl-β-D-glucosamine, and *N*-acyl-β-D-glucosamine^2,3^. For teicoplanin, there are six major subtypes (A2-1 through A2-5, and A3-1) and four minor subtypes (from RS-1 to RS-4)^4^. Of these subtypes, teicoplanin is primarily formed by bacteria as a blend of A2-1 through A2-5 lipoforms which have different fatty acid chains attached to the glcNAc (N-acetyl-β-D-glucosamine) residue^5^. The antibiotic mechanism of teicoplanin is similar to another glycopeptide vancomycin (structurally similar, although not containing lipid), and both antibiotics inhibit the formation of the peptidoglycan chains of bacterial cell walls, by attaching to the D-Ala-D-Ala C-terminus of the pentapeptide substrate via hydrogen bonds^6^. Moreover, teicoplanin is known to interact with this pentapeptide substrate through its hydrophobic lipid chain, resulting in the positioning of the antibiotic being adjacent to the peptidoglycan^7,8^.

Teicoplanin is used in the treatment of life-threatening infectious diseases caused by multidrug-resistant Gram-positive bacteria, including methicillin-resistant *Staphylococcus aureus* (MRSA) and *Enterococci*. Teicoplanin has a proven, outstanding high efficacy in various tissue sites, such as the heart and respiratory tracts^9^. Its main routes of administration are intravenous and intramuscular, although it is also given orally and is considered for topical administration, especially for treating ocular infections.

The therapeutic plasma concentration of teicoplanin ranges from 10 to 30 mg/L, depending on the severity of the disease or the range of infectious sites, for example, bone infections^10–12^, and has been found to bind to serum albumin in the blood^10^. On the other hand, oral and topical (ocular infections) administrations are limited. The oral route is used for the treatment of pseudomembranous colitis caused by *Clostridium difficile*^13^. In terms of ocular infections, Kaye suggested synergistic benefits of teicoplanin with other antibiotics, such as meropenem, against *S. aureus* keratitis^14,15^, although Kaye’s research group concluded that there was little penetration of teicoplanin into human aqueous humour below the cornea with the administration of 10 mg/mL eye drops^16^. Antoniadou et al. also reported a similar result: no penetration into the aqueous humour, with the subconjunctival injection (approximately 0.5 mL) of 25 mg teicoplanin^17^.

Since the beginning of the Covid-19 pandemic in December 2019, teicoplanin has been spotlighted as a potential drug candidate against severe acute respiratory syndrome coronavirus 2 (SARS-CoV-2) due to its well-known antiviral ability. Zhou and colleagues had earlier indicated that teicoplanin inhibited cell entry of the SARS-CoV virus^18^. In order to cross the cell membrane and enter a host cell, both SARS-CoV and SARS-CoV-2 viruses depend on cysteine proteinase cathepsin L (CTSL), which splits viral spike (S) glycoproteins attached to a host receptor so that viruses are released from an endosome within the host cell^18,19^. The fatty acid chain of teicoplanin interacts with CTSL, while vancomycin, without such a hydrophobic group, cannot express antiviral activity against CTSL-dependent viruses^18^. Consequently, some clinical studies were focused on the novel medical use of teicoplanin as a Covid-19 drug^20,21^. Regardless of whether teicoplanin is used in the treatment of Covid-19 or co-infections of Gram-positive bacteria in Covid-19 patients, its use is still in demand.

However, there has been a growing concern about the resistance to teicoplanin in pathogens since its approval in Europe in 1988. In the same year, it was already shown that vancomycin- and teicoplanin-resistant *Enterococci* strains had been isolated from patients in France^22^. Gram-positive bacteria, especially *Enterococci*, acquire resistance by modifying their D-Ala-D-Ala moiety of peptidoglycan precursors, Lipid II. This moiety is transformed to either D-Ala-D-Lac (*vanA, vanB, vanD*) or D-Ala-D-Ser (*vanC, vanE, vanG*) in resistant strains, and as a result, glycopeptides have a low affinity to these phenotypes of precursors^23,24^.

Although it is important to explore how teicoplanin binds to the Lipid II moiety regardless of its phenotypes, the knowledge of the biological form of teicoplanin in an aqueous solution is of importance. Teicoplanin and its aglycon derivatives^25^ use their long acetyl chain to attach themselves to the targeted sites. It is known that the minimum volume of solvent required to dissolve 400 mg of teicoplanin is 3 ml because, below that value, a gel might be formed in solution^10^. Teicoplanin derivatives have also been reported to create nano-sized aggregates in aqueous solution^26^. This gelation/coalescence of teicoplanin was thought to be caused by micellization due to its hydrophobic tail^27,28^, although a teicoplanin derivative without that tail can still aggregate in solution^29^. It is hypothesized that aggregation enables teicoplanin to have enhanced binding potency^30^. However, this concentration-dependent aggregation might lead to poor permeability of teicoplanin across the epithelial lining by the oral and topical (ocular) routes and the aggregated form may reduce effective concentrations on sites, resulting in the need for a greater dose and then further bacteria acquiring resistance^31^. Due to its importance, in this study, we perform an analysis of this associative/aggregation effect using the powerful hydrodynamic techniques of sedimentation velocity and sedimentation equilibrium in the analytical ultracentrifuge (SV-AUC, SE-AUC) taking advantage of the inherent separation and analysis facilities of the analytical ultracentrifuge (AUC). Analytical ultracentrifugation is a matrix-free method with a broad range of molar masses, 10^2^–10^8^ g/mol, and the key technique used to explore the molecular behaviour of proteins, polysaccharides, or other macromolecules in solution^32^. AUC has recently been used to characterize the self-associative properties of vancomycin^33^ and its interactions with VanS^34^ and mucins^35^.

We then also assess the solution conformation of the association/aggregation products using molecular viscometric analysis of the intrinsic viscosity [$\eta$], in combination with the sedimentation coefficient from sedimentation velocity. We believe this present study is the first report demonstrating the self-association of teicoplanin with hydrodynamic methods.

## Materials and methods

### Teicoplanin

Teicoplanin A2 (monomer molar mass: *M*_1_ = 1877.6 g/mol for teicoplanin A2-1, *M*_1_ = 1879.7 g/mol for teicoplanin A2-2 and A2-3, and *M*_1_ = 1893.7 g/mol for teicoplanin A2-4 and A2-5) was purchased in powder form from Sigma-Aldrich, United Kingdom. Its structure is shown in Fig. 1:

Teicoplanin lipoform A2-2 (*M*_1_ = 1879.7 g/mol) is shown: the other major lipoforms of A2 with different acyl chains are shown in the inset.

Teicoplanin samples were prepared in a phosphate-chloride buffered saline solution (PBS, or “Paley buffer”) at pH ~ 6.8 and, by adding NaCl, adjusted to an ionic strength of *I* = 0.1 mol/L^38^.

The concentration, *c* (g/mL) of the stock solution was then measured using a differential refractometer (Atago DD7, Tokyo, Japan) set to zero with the reference solvent (PBS) and using a refractive increment *dn/dc* of 0.188 mL/g for teicoplanin^39^. The measured concentration was multiplied by 0.96 for moisture content correction, being calculated from the difference in the weights of teicoplanin powder before and after the vacuum oven (Vacuum Oven 31 L, Fistreem, Cambridge, UK) drying overnight^40^.

The partial specific volume $\overline{\upsilon }$ from solution/ solvent densities was determined using an Anton-Paar (Graz, Austria) digital density meter^41^, and application of:

1 $$\overline{\upsilon } = \frac{1}{{\rho_{0} }} \cdot \left( {1 - \frac{{\rho - \rho_{0} }}{c}} \right)$$

at a concentration, *c*, of 10.2 mg/mL, and where $\rho$ and $\rho_{0}$ are the densities of the solution and solvent, respectively. A value of (0.64 ± 0.01) mL/g, was obtained, similar to that for vancomycin^33^.

### Sedimentation velocity in the analytical ultracentrifuge

Experiments to determine sedimentation coefficients and sedimentation coefficient distributions were performed at a temperature of 20.0 °C (at which standardised values are easily calculated) using an Optimal XL-I analytical ultracentrifuge (Beckman Instruments, Palo Alto, CA, USA) with Rayleigh interference optics. Teicoplanin samples (400 $\mu L$) and reference solvent (PBS, 420 $\mu L$) were injected into channels of the 12 mm double sector epoxy cells with sapphire windows. These cells were then centrifuged at 47,500 rpm for a run time of ~ 24 h and the data obtained were analysed in SEDFIT using the least squares, ls-g*(*s*) processing method^42^. This generates the sedimentation coefficient distribution, g(*s*) versus $s_{{{\text{T}},{\text{b}}}}$, where *s*_T,b_ is the sedimentation coefficient, at temperature *T* in buffer *b*. The *s* value in Svedberg units, *S* = $10^{ - 13}$ seconds, was then normalised to standard conditions (density $\rho_{20,w}$ and viscosity $\eta_{20,w}$ of water at 20.0 °C) to give $s_{20,w}$ from the Eq. ^43^:

2 $$s_{20,w} = \frac{{1 - \overline{{\upsilon { }}} \cdot \rho_{20,w} }}{{1 - \overline{{\upsilon { }}} \cdot \rho_{T,b} }} \cdot \frac{{\eta_{T,b} }}{{\eta_{20,w} }} \cdot { }s_{T,b}$$

where $\rho_{T,b}$ and $\eta_{T,b}$ are the density and the viscosity of buffer *b* at temperature *T*, respectively.

### Sedimentation equilibrium in the analytical ultracentrifuge

Sedimentation equilibrium experiments were used to obtain equilibrium concentration distribution profiles for absolute molecular weight measurement. An Optima XL-I analytical ultracentrifuge was also employed but at a lower temperature of 7 °C because of the longer duration of a sedimentation equilibrium experiment compared to sedimenation veloicity. To characterise the self-association/aggregation of teicoplanin, 12 mm double sector epoxy cells were loaded with the same volumes (100 $\mu L$) of both solution and solvent and run at 45,000 rpm for a run time of ~ 48 h. Records of concentration distributions of teicoplanin at equilibrium were subsequently analysed using the model-independent SEDFIT-MSTAR algorithm^44^. Since the non-ideality of teicoplanin is negligible we estimated that apparent weight average molar masses $M_{w,app}$ were approximately equal to the true weight average molar masses $M_{w}$^45^. The multiple concentrations used effectively represent repeats for the sedimentation equilibrium (and sedimentation velocity) experiments.

### Hydrodynamic radius determination by dynamic light scattering (DLS)

Dynamic or quasi-elastic light scattering (DLS or QLS) measurements were made on the fixed scattering angle Zetasizer Nano-S system (Malvern instruments Ltd., Malvern UK) equipped with a 4mW He–Ne laser at a wavelength of 632.8nm^46,47^. Samples in solution were measured in a quartz cuvette at 20.0 °C. A scattering angle of 173° was used, and collected in manual mode, requiring a measurement duration of 90 s, averaged over several measurements. The resulting data were analysed using the “Zetasizer Software (Version 7.1)” (Malvern Instruments Ltd., Malvern, UK), providing a volume distribution of translational diffusion coefficients based on a form of the CONTIN program^48^. The viscosity of the buffer used was calculated using a solvent builder interface and takes the effects of buffer salts into account. The z-average hydrodynamic radii *r*_*z*_ (nm), were evaluated from the z-average translational diffusion coefficients *D*_z_ by the Stokes–Einstein Eq. ^47^:

3 $$r_{{\text{z}}} = {\text{ k}}_{{\text{B}}} T/\{ {6}\pi \eta_{{\text{o}}} D_{{\text{z}}} \}$$

where k_B_ is the Boltzmann constant, *T* is the absolute temperature and η is the viscosity of the medium. The following assumptions were made (i) the solutions were sufficiently dilute and sample sizes sufficiently small that non-ideality effects were not significant—i.e. an extrapolation to zero concentration was not necessary. This is reasonable as the non-ideality is due to the low concentration of mucin and small size of teicoplanin, and for translational diffusion, the two main contributory factors to non-ideality—the hydrodynamic and thermodynamic terms—compensate for each other and can even cancel each other out^49,50^. (ii) the teicoplanin in its monomeric and multi-meric form were quasi-spheroidal and not asymmetric so there was no angular dependence of the measured *D*_z,_ values on anisotropic rotational diffusion effects—i.e. an extrapolation to zero angles was not necessary.

### Intrinsic viscosity measurement

Teicoplanin solutions were analysed using the capillary viscometer AMVn (Anton-Paar, Graz, Austria). This measurement was conducted at a temperature of 25.0 °C based on the rolling ball viscosity method. With a 1.4 mm steel ball moving in a 1.6 mm diameter glass capillary, the flow times (averaged over repeat measurements) of the solvent and solution were then determined. The relative viscosity was calculated from the equation:

4 $$\eta_{rel} = \frac{\rho \cdot t}{{\rho_{0} \cdot t_{0} }} = \eta_{sp} + 1$$

where $\eta_{sp}$ is the specific viscosity^51^. Then the intrinsic viscosity $\left[ \eta \right]$ was estimated from the Solomon-Ciuta relation^52^ at a concentration of 10.2 mg/ml, which gave a sufficient flow time increment between solvent and solution:

5 $$\left[ \eta \right] \approx \frac{1}{c} \cdot \left[ {2\eta_{sp} - 2ln\left( {\eta_{rel} } \right)} \right]^{\frac{1}{2}}$$

## Results and discussion

### Hydrodynamic properties of teicoplanin

**Figure 2:**
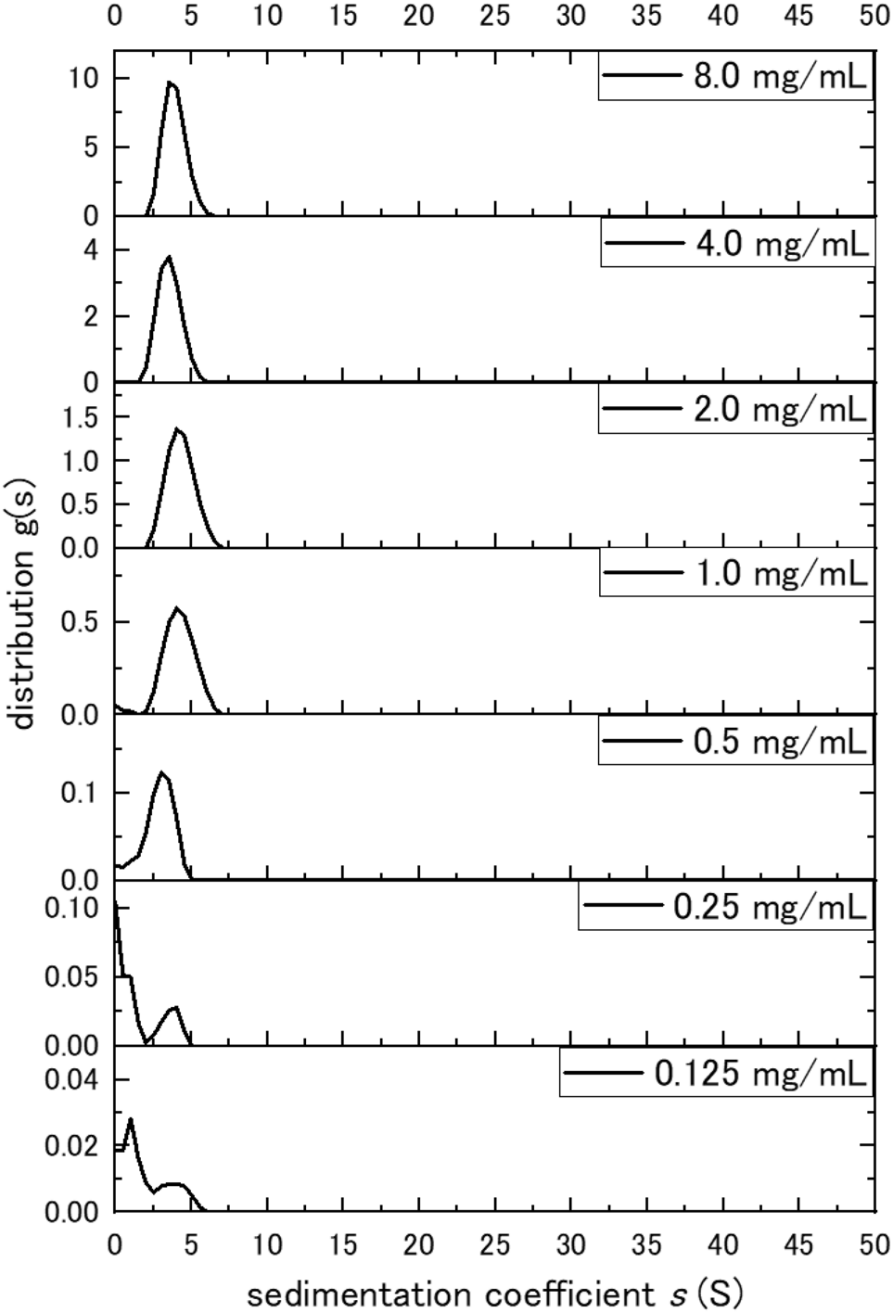
Sedimentation coefficient distribution of teicoplanin A2 at different concentrations from 0 to 5 mg/mL. The Y-axis ranges are different for each sample because that is clearer to see unimodality for higher concentrations (> 0.5 mg/mL) and separation occurring for lower concentrations (< 0.5 mg/mL).

**Figure 3:**
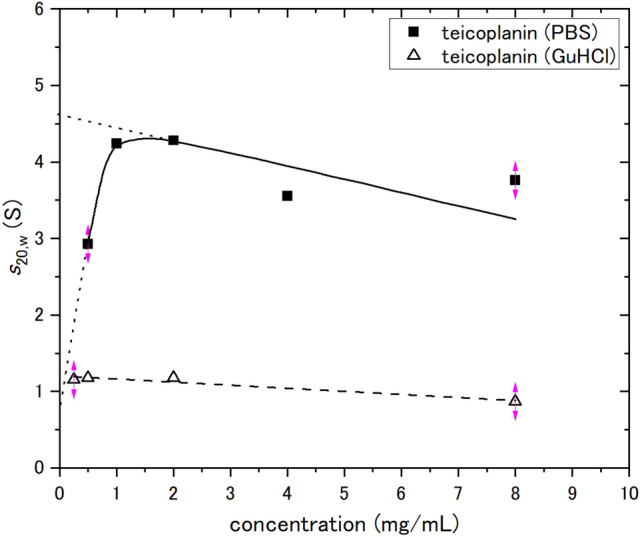
Change of apparent sedimentation coefficient (*s*_20,w_) of teicoplanin A2 with sedimenting concentration, *c*. Concentrations were corrected for radial dilution. The extrapolated value of ~ 4.65 S is consistent with a spherical 18–19-mer of molar mass of 35,400 g/mol. The lower extrapolated value of ~ 0.7 S is the predicted value for a spheroidal unimer, which is consistent with the *s*-value of teicoplanin dissolved in 6 M GuHCl, *s*_20,w_ = (1.17 ± 0.01) S. Solid line is a standard French curve fit to the data.

Figure 2 shows the sedimentation coefficient distribution function g(*s*) plotted versus *s*_20,w_, where g(*s*) is the proportion of sedimentation coefficient values lying within the range of* s* and *s* + *ds*. Sedimentation velocity plots obtained using the algorithm SEDFIT for teicoplanin (Fig. 2) reveal unimodal behaviour at much higher *s*-values than expected for monomeric teicoplanin, for concentrations > 0.5 mg/ml. Below this concentration separation occurred with bimodality. Fig. 3 shows a plot of *s* vs *c* for those concentrations where unimodality is still clear. The extrapolated value of ~ 4.65 S is in good agreement with a spherical 18–19-mer of the molar mass of 35,400, while the lower extrapolated value of ~ 0.7 S is the predicted value for a spheroidal unimer.

**Figure 4:**
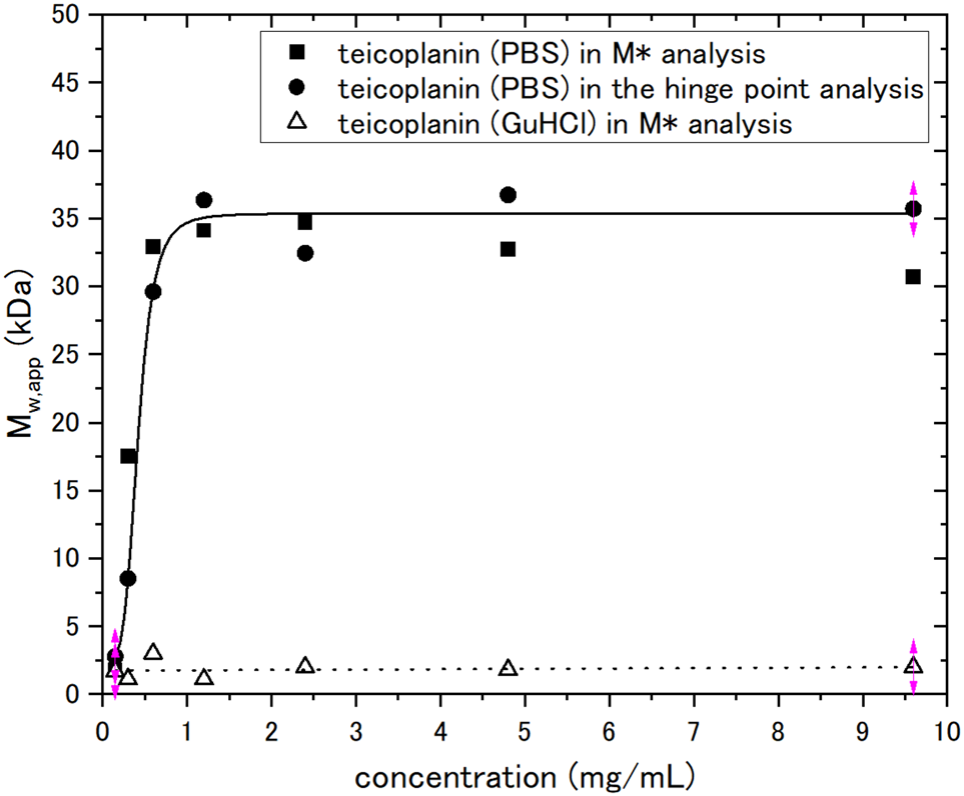
Change of weight average molar mass *M*_*w*_ of teicoplanin with loading concentration derived from sedimentation equilibrium analysed by SEDFIT–MSTAR. Solid square symbols are molar masses *M*_*w,app*_ obtained from the M* method. Solid round symbols are molar masses* M*_*w,app*_ obtained from the hinge point method. Solid line is a standard French curve fit to the data. Open triangle symbols are molar masses *M*_*w,app*_ of teicoplanin dissolved in 6 M GuHCl from the M* method.

In order to effectively assess the 19-merisation, we sought the application of a chaotropic agent (6 M GuHCl) to reduce the solvent effects of water and make teicoplanin more soluble. The *s*-value of teicoplanin dissolved in 6 M GuHCl was ~ 0.7 S and *s*_20,w_ was (1.17 ± 0.01) S. For the weight-average molar mass, *M*_w_ = (1.75 ± 0.35) kDa (see Fig. 4), corresponding the unimer, was obtained. Since teicoplanin was dissolved in the chaotropic agent, the teicoplanin samples did not become 18–19mer over 0–10 mg/mL.

### Teicoplanin self-association

To determine the weight-average molar masses $M_{w}$ of teicoplanin, the M* extrapolation method^45^ and hinge point analysis^44^, both incorporated in the sedimentation equilibrium-based SEDFIT–MSTAR software^44^ were used. A similar approach was previously used in the analysis of vancomycin^33^. Rayleigh interference optics provides an accurate record of a sedimentation equilibrium concentration profile *c(r)* vs *r*, which means that the local concentration *c(r)* at the radial position *r* (cm) is from the rotation centre. M*(*r*) is a useful operational point average molar mass parameter. M*(*r* → *r*_b_) = *M*_w_ is the weight average molar mass over the whole macromolecular distribution, where *r* is the radial position at the cell base. This method is particularly advantageous for polydisperse/ or self-associating systems^45^. As an additional check, the “hinge point method” (the value of the point weight average molar mass, *M*_w_(*r*) at the “hinge point” in the sedimentation equilibrium distribution, i.e. the radial position in the cell where the local concentration *c*(*r*) = the original loading concentration) provides another estimate for the whole distribution molar mass *M*_w_^44^.

When the value of apparent weight average molar masses *M*_w_ were extrapolated to zero concentration a value of (1.9 ± 0.1) kDa was obtained, comparable to (*M*_1_ = 1879.7 g/mol) of teicoplanin A2-2. The SEDFIT-MSTAR algorithm also gives an approximation of the point weight average molar mass at the hinge point *r*_*hinge*_. At this radial position *r*_*hinge*_ the corresponding concentration *c(r)* is equal to the initial loading concentration *c*, *M*_*w*_*(r*_*hinge*_*)* = *M*_*w*_. The value of (2.7 ± 0.1) kDa was obtained—higher compared with a monomer molar mass of teicoplanin A2-2 because of self-association.

Regardless of whether using the hinge point method or the M* method, the change of the apparent weight average molar masses *M*_*w,app*_ plateaus from *c* = 1 mg/mL (Fig. 4), giving a value of (35,400 ± 1000) g/mol. This corresponds to ~ 19mers in the hinge point method while (33,000 ± 1000) g/mol corresponds to ~ 18mers for the M* method.

### Dynamic light scattering analysis

**Figure 5:**
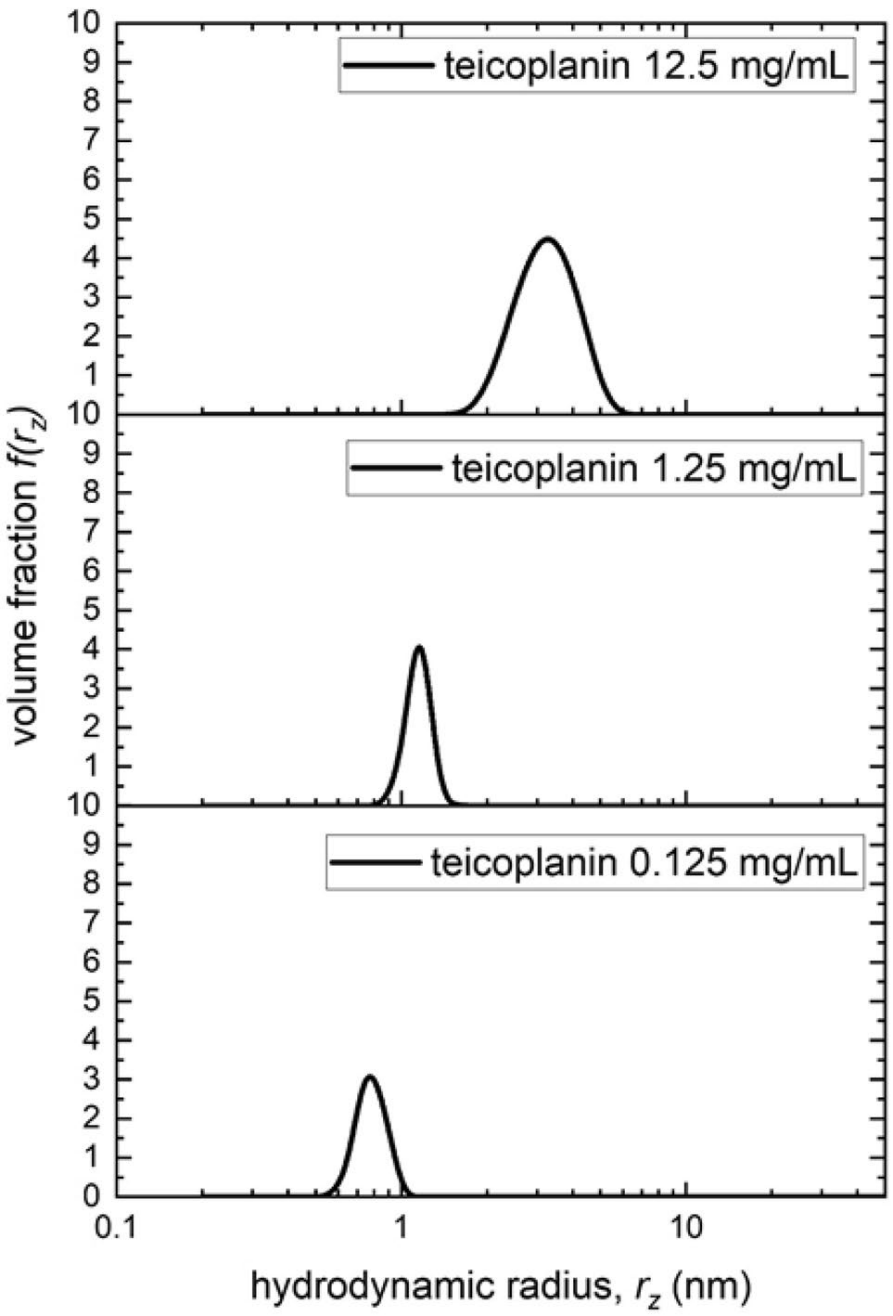
Distribution of z-average hydrodynamic radii obtained from dynamic light scattering measurements at 20.0 °C for teicoplanin in solution at concentrations 12.5, 1.25 and 0.125 mg/mL.

The self-associative process was confirmed by DLS measurements. Three concentrations were analysed (0.125, 1.25 and 12.5 mg/mL). At 12.5 mg/mL (which corresponds from Figs. 3 and 4 to the 18–19 mer species) a particle size *r*_z_ ~ 3.2 nm is observed, and as the concentration is lower the size distribution becomes clearly smaller, indicating dis-assembly towards a smaller particle (Fig. 5).

### Conformational analysis of teicoplanin 18–19mer assembly

The sensitive hydrodynamic conformation probe of intrinsic viscosity [$\eta$] was used to assess the conformation of the teicoplanin ~ 19mer assembly, reinforced by the sedimentation coefficient, molar mass and (z-averaged) hydrodynamic radius *r*_z_ from dynamic light scattering. To avoid possible dissociation effects and to ensure a sufficient flow-time increment, we estimate [$\eta$] using the Solomon-Ciuta Eq. (5) at a concentration of 10.2 mg/mL. A value for [$\eta$] of (3.2 ± 0.1) mL/g is obtained.

In order to interpret this in terms of a molecular shape account needs to be taken of the contribution of the swollen specific volume of the assembly in solution ${\text{v}}_{{\text{s}}}$ (which will be swollen due to a time-averaged association with the surrounding solvent through dynamic hydrogen bonding and other associative processes)^43^:

6 $$[\eta ] \, = \nu .{\text{v}}_{{\text{s}}}$$

**Figure 6:**
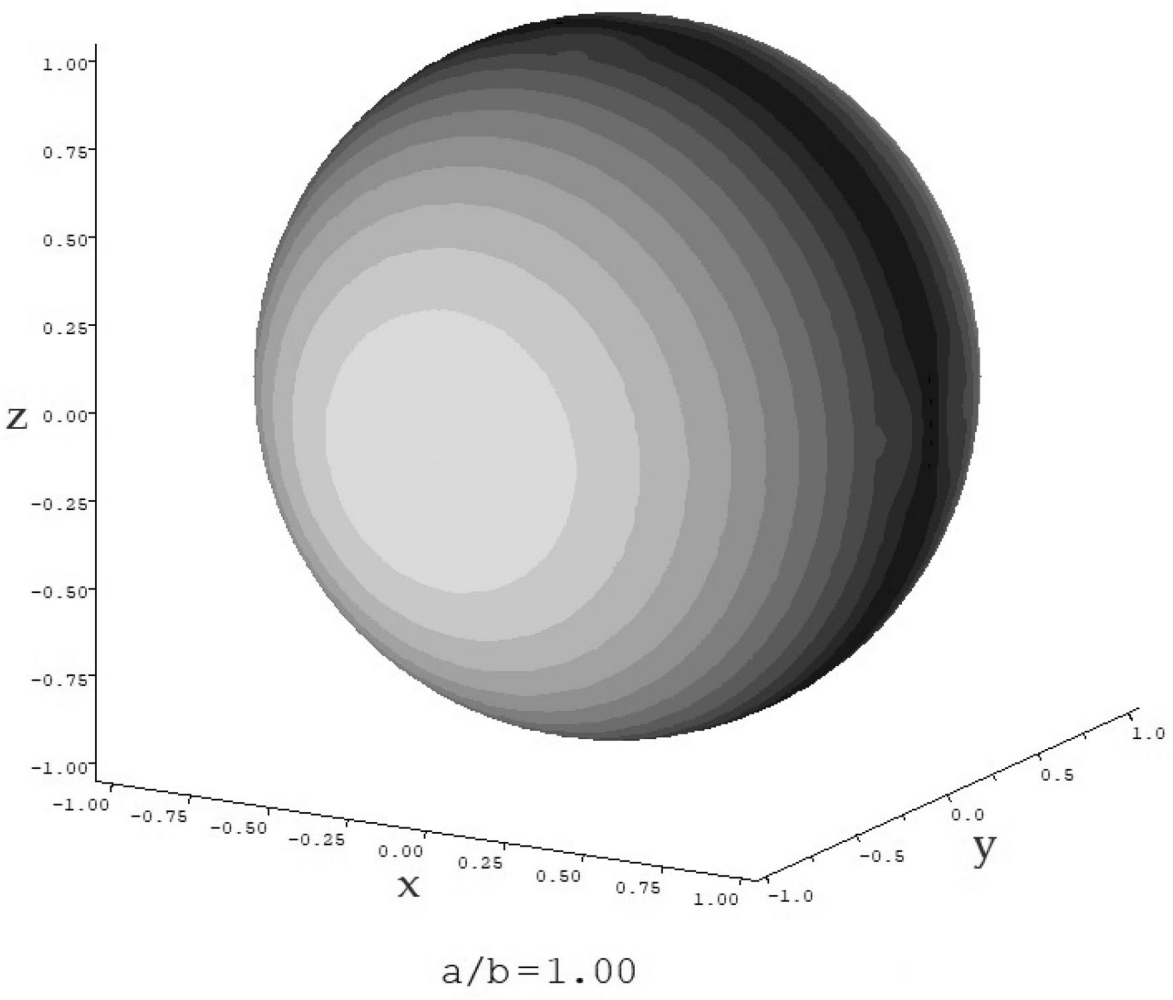
ELLIPS1^53^ representation of the conformation of teicoplanin showing an axial ratio (*a/b*) = 1: i.e. a sphere.

Equation (6) $\nu$ is the Einstein-Simha shape factor. v_s_ is likely to be higher for the glycopeptide than for proteins due to the relatively large proportion of carbohydrate which tends to have a greater affinity for solvent. In Table 1 values of the shape factor $\nu$, and their corresponding ellipsoid of revolution axial ratios *a/b* were calculated based on either a prolate or oblate model, using the routine ELLIPS1 (Harding et al. 1997) for 3 cases of v_s_/${\overline{\upsilon }}$ , including the (unlikely) case of no swelling v_s_/${\overline{\upsilon }}$ = 1. The maximum value for v_s_/${\overline{\upsilon }}$ ~ 2, which corresponds to the minimum value of *a/b* = 1 (i.e. a sphere, Fig. 6), and this seems the most likely scenario.

**Table 1:** Values of the viscosity shape function $\nu$ and axial ratio *a/b* for different values of v_s_.

v_s_/${\overline{\upsilon }}$	v_s_ (mL/g)	$\nu$	(*a/b*) prolate	(*a/b*) oblate
1*	0.64	5.0	4.3	5.4
1.5	0.96	3.3	2.5	2.8
2	1.28	2.5	1	1

*no swelling through dynamic hydration effects. v_*s*_ swollen specific volume, ${\overline{\upsilon }}$ partial specific volume (0.64 mL/g).

**Figure 7:**
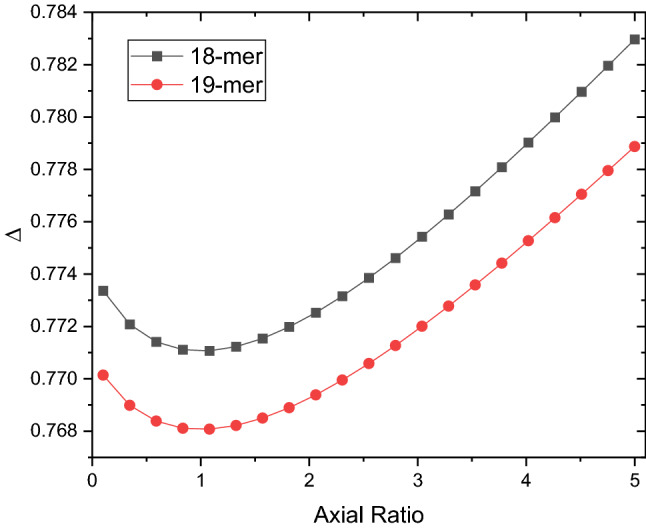
Minimisation function performed by SingleHYDFIT on teicoplanin, using the above hydrodynamic parameters and molar mass consistent with either 18-mer (black square) or 19-mer (red circle). Both plots minimise to an axial ratio of 1.0.

To check this, we use a global fitting approach known as SingleHYDFIT^54^ which combines intrinsic viscosity data with sedimentation coefficient and dynamic light scattering (hydrodynamic radius) data together, along with the molecular weight and partial specific volume. It involves the minimization of a global fitting function (Fig. 7). The E2 protocol (ratio of ellipsoid) was chosen and run twice: once with an assumed molar mass equivalent of an 18-mer (33835 Da) and once again with an assumed molar mass of 19-mer (35714 Da). Delta (Δ) was plotted against axial ratio, where values < 1 mean oblate and > 1 mean prolate (Fig. 7).

The plot shows an optimisation of axial ratios, providing an indication of most likely value to occur. SingleHYDFIT yielded an axial ratio of (1.0 ± 0.0) for both molar masses, suggesting that the supramolecular structure is that of a sphere regardless of whether it is 18- or 19-mer, and confirming the swelling factor of 2 through hydration.

## Concluding remarks

In conclusion, based on the matrix-free methods of analytical ultracentrifugation and macromolecular viscometry, teicoplanin appears in phosphate-chloride buffered solution at pH6.8 and *I* = 0.10 mol/L as a spheroidal 18–19mer assembly with a swelling ratio in solution of ~ 2 which dissociates at concentrations < 0.5 mg/mL. This spherical conformation would be consistent with a micellar-like association with the acyl chains on the inside.

There are some similarities with another “last line of defence” glycopeptide antibiotic vancomycin^33,55^ On the one hand, vancomycin also shows reversible self-associative behaviour above a similar concentration, but this appears to largely truncate to a monomer–dimer only. On the other hand, teicoplanin with its higher degree of glycosylation (two residues in vancomycin versus three residues in teicoplanin) and the lipid chains attached to one of the glcNAc residue self-associates to give a much larger 18–19mer structure in solution, a structure which is broken by the hydrogen bond and ionic bond disruptive agent 6 M GuHCl. As to the nature of this large spherical n-mer association, it could either be due to micellization inspired by its fatty acid chain^27,28^ or it could be due to the non-specific association of its other hydrophobic regions^29^. Interestingly, *teicoplanin aglycon* without a lipid chain had previously been found to dimerise weakly in solution^29^. Each or both structural differences may affect the number of building blocks during polymerisation, requiring further research by sedimentation equilibrium experiments using teicoplanin derivatives without either a long acyl chain or sugar units.

In the clinical setting, serum concentrations of teicoplanin are ~ 10 μg/mL or 10 mg/L for intravenous injection^10^, resulting in a unimer form of teicoplanin in blood, pH7.4 and *I* = 0.15 mol/L^56^. On the other hand, 10 mg/mL of eye drops^16^ would lead to the 18–19 mers on the conjunctiva, which makes it harder for teicoplanin to permeate beyond the cornea. This means that any concentration > 0.5 mg/mL has the potential to reduce topical penetration, while at lower concentrations (< 10 mg/L or 10 μg/mL) the dose is below the therapeutic concentration and thus ineffective^10^. Furthermore, if the hydrophobic acyl groups are involved with the binding to the bacterial peptidoglycan, then micellization would appear to reduce the efficacy as an antibiotic at these higher doses.

Additionally, the methods we used can be applied to other members of glycan antibiotics, such as dalbavancin, a lipoglycopeptide with both a fatty acid chain and two sugar residues. Dalbavancin is a second-generation drug developed based on both vancomycin and teicoplanin^57^. Furthermore, we could examine the degree of polymerisation of eremomycin, another glycopeptide antibiotic, over clinically available concentrations to check its high-order oligomeric states reported by an NMR study^58^. Our combined understanding of the different hydrodynamic behaviour of vancomycin and teicoplanin will help develop important future-generation antibiotic drugs, resulting in a better understanding of the structural effects on the aggregational behaviour of some antibiotics. The presence of the third carbohydrate residue and its reinforcement of potential hydrophobic interactions of teicoplanin also bears comparison with a new study using molecular dynamics simulations of the semisynthetic disaccharide antibiotic oritavancin which opens the door for a new generation of antibiotics in the fight against bacterial disease^53^—and the increasing threat of antimicrobial resistance^59,60^.

## Data Availability

The datasets used and analysed in the current study are available from the corresponding author upon reasonable request.
